# 1647. Clinical Outcomes in Pediatric Appendectomy Patients Treated with Piperacillin-Tazobactam versus Ceftriaxone/Metronidazole

**DOI:** 10.1093/ofid/ofad500.1481

**Published:** 2023-11-27

**Authors:** Reese Cosimi, Florian Daragjati, Sasha Ward, Maria Whitmore, Fernandez Marisol, Ana Cristina Perez Moreno, Collin Miller, Mohamad G Fakih

**Affiliations:** Ascension, Indianapolis, Indiana; Ascension, Indianapolis, Indiana; Ascension Sacred Heart Pensacola, Pensacola, Florida; Peyton Manning Children's Hospital at Ascension St. Vincent, Indianapolis, Indiana; Dell Children's Medical Center of Central Texas, Dell Medical School and UT Austin, Austin, Texas; Ascension Health, Franklin, Wisconsin; Ascension Health, Franklin, Wisconsin; Ascension, Indianapolis, Indiana

## Abstract

**Background:**

Appendicitis is the most common cause of emergent pediatric surgeries. The standard treatment includes surgical removal of the appendix plus or minus antibiotic therapy. Piperacillin-tazobactam or ceftriaxone/metronidazole are commonly utilized antibiotic regimens. While the latter option may be preferable for antimicrobial stewardship measures due to its narrower spectrum of activity, current existing evidence is conflicting as to whether one results in improved clinical outcomes.

**Methods:**

Retrospective cohort study across four regionally diverse pediatric hospitals of all admitted patients aged 0 to 17 years with a procedure code for appendix resection between January 1, 2021 to December 31, 2022. Patients were included if they received either piperacillin-tazobactam or ceftriaxone/metronidazole during the encounter corresponding to the appendectomy procedure. Outcomes measured include length-of-stay (LOS) and 30-day readmission.

**Results:**

A total of 600 appendectomy cases were included in the analysis of which 463 received ceftriaxone/metronidazole and 137 patients received piperacillin-tazobactam. Patients aged < 10 years old were more likely to receive piperacillin-tazobactam. More perforated appendicitis cases were noted in the patients who received ceftriaxone/metronidazole. Average LOS in days (standard deviation) was not different between the two groups, 4.2 (6.1) and 3.9 (6.3), respectively (*p*=0.25). Readmissions were more common in the patients who received piperacillin-tazobactam, 3.2% and 7.3% (*p*=0.04).

**Figure:**
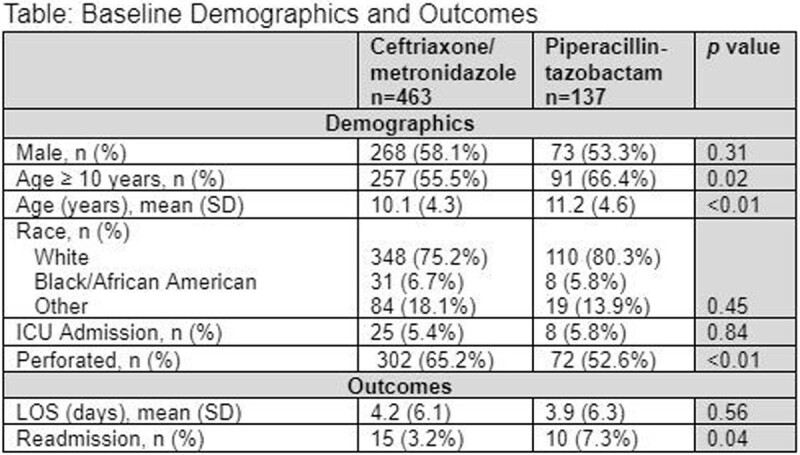

**Conclusion:**

Clinical outcomes in pediatric appendectomy patients do not appear to be associated with antibiotic regimen selection. Our study supports the continued use of ceftriaxone/metronidazole first line for appendicitis treatment in children taking into account local susceptibility patterns.

**Disclosures:**

**Reese Cosimi, PharmD**, Allergen: Advisor/Consultant

